# Supplementary material for: Anti-Müllerian Hormone Levels among Female Firefighters
Source: Int J Environ Res Public Health. 2022 May 14;19(10):5981. doi: 10.3390/ijerph19105981 (PMC9141260; doi:10.3390/ijerph19105981)
Supplement: Supplementary file 1 [file ijerph-19-05981-s001.zip › ijerph-1646144-supplementary.pdf]

Table S1. Sensitivity Analysis Percent Change in Anti-müllerian Hormone Levels Comparing Non-Firefighters and Firefighters, Excluding Probably Polycystic Ovary Syndrome (*n*=142).

Firefighting Status	AMH (ng/mL), Mean ± SD, [Range]	Model 1 % Difference (95% CI)	Model 2 % Difference (95% CI)
Non-firefighters (*n*=50)	2.89 ± 2.56, [0.26, 9.70]	0.00 (Ref.)	0.00 (Ref.)
Firefighters (*n*=92)	1.96 ± 2.06, [0.02, 8.48]	-56.78 (-74.88, -25.62)	-34.46 (-56.40, -1.46)

Abbreviations: AMH- Anti-müllerian hormone; CI- Confidence Interval; SD- Standard Deviation

Model 1: Linear regression of log(AMH) and firefighting status

Model 2: adjusted for age, age^2, and Body Mass Index

% Difference Calculated by: ([exp(β) −1] × 100)

Table S2. Sensitivity Analysis Among Firefighters, Occupational Exposures and Percent Change in Anti-müllerian Hormone, Excluding Probable Polycystic Ovary Syndrome (*n*=92).

Firefighting-Related Exposure	AMH (ng/mL), Mean ± SD, [Range]	Model 1 % Difference (95% CI)	Model 2 % Difference (95% CI)
Years in the fire service continuous (per 5-year increase)		-51.14 (-60.77, -39.15)	1.06 (-25.79, 37.62)
Live fires responded to in a typical month		2.39 (-4.33, 9.58)	2.20 (-2.63, 7.27)
Poor fit of any personal protective equipment			
No (*n*=43)	1.88 ± 2.03, [0.02, 7.21]	0.00 (Ref.)	0.00 (Ref.)
Yes (*n*=49)	2.03 ± 2.11, [0.02, 8.48]	25.40 (-39.82, 161.28)	62.74 (-2.92, 172.80)
Poor fit of self-contained breathing apparatus (SCBA)			
No (*n*=70)	1.92 ± 2.04, [0.02, 8.48]	0.00 (Ref.)	0.00 (Ref.)
Yes (*n*=22)	2.08 ± 2.16, [0.02, 8.09]	22.11 (-48.30, 188.41)	57.63 (-15.89, 195.39)
Wildland firefighting			
No (*n*=60)	2.04 ± 2.13, [0.02, 8.09]	0.00 (Ref.)	0.00 (Ref.)
Yes (*n*=31)	1.74 ± 1.92, [0.02, 8.48]	3.64 (-52.44, 125.83)	-11.72 (-49.21, 53.46)

Abbreviations: AMH- Anti-müllerian hormone; CI- Confidence Interval; SD- Standard Deviation

Model 1: Linear regression of log(AMH) and firefighting-related exposure

Model 2: adjusted for age, age^2, and Body Mass Index

% Difference Calculated by: $([\exp(\beta) - 1] \times 100)$

Table S3. Percent Change in Anti-müllerian Hormone Levels Comparing Non-Firefighters and Firefighters, Stratified by Age Group (*n*=164).

Age Group	Firefighting Status	AMH (ng/mL), Mean ± SD, [Range]	Model 1 % Difference (95% CI)	Model 2 % Difference (95% CI)	Interaction *p*-Value*
<32 years (*n*=40)	Non-firefighters (n=18)	6.70 ± 5.40, [0.47, 17.3]	0.00 (Ref.)	0.00 (Ref.)	0.71
	Firefighters (n=22)	5.56 ± 4.62, [1.25, 17.3]	-2.15 (-46.63, 79.38)	-1.62 (-47.13, 83.09)	
32 to 37 years (*n*=46)	Non-firefighters (n=19)	4.82 ± 3.76, [0.36, 13.59]	0.00 (Ref.)	0.00 (Ref.)	
	Firefighters (n=27)	4.76 ± 4.57, [0.13, 15.37]	-17.25 (-57.54, 61.25)	-16.20 (-58.29, 68.37)	
38+ years (*n*=78)	Non-firefighters (n=21)	1.98 ± 3.03, [0.16, 14.08]	0.00 (Ref.)	0.00 (Ref.)	
	Firefighters (n=57)	1.05 ± 1.39, [0.02, 6.77]	-63.51 (-83.77, -17.93)	-49.17 (-74.73, 2.25)	

*Interaction term between age category and firefighting status

Model 1: Linear regression of log(AMH) and firefighting-related exposure

Model 2: adjusted for age, age^2, and Body Mass Index

% Difference Calculated by: ([exp(β) −1] × 100)
